# Neuroimaging as a Window Into the Pathophysiological Mechanisms of Schizophrenia

**DOI:** 10.3389/fpsyt.2021.613764

**Published:** 2021-03-11

**Authors:** Nina Vanessa Kraguljac, Adrienne Carol Lahti

**Affiliations:** Neuroimaging and Translational Research Laboratory, Department of Psychiatry and Behavioral Neurobiology, University of Alabama at Birmingham, Birmingham, AL, United States

**Keywords:** pharmacological challenge, treatment response, duration of untreated psychosis, functional MRI, magnetic resonance spectroscopy, diffusion weighted imaging, first episode psychosis, antipsychotic naïve

## Abstract

Schizophrenia is a complex neuropsychiatric disorder with a diverse clinical phenotype that has a substantial personal and public health burden. To advance the mechanistic understanding of the illness, neuroimaging can be utilized to capture different aspects of brain pathology *in vivo*, including brain structural integrity deficits, functional dysconnectivity, and altered neurotransmitter systems. In this review, we consider a number of key scientific questions relevant in the context of neuroimaging studies aimed at unraveling the pathophysiology of schizophrenia and take the opportunity to reflect on our progress toward advancing the mechanistic understanding of the illness. Our data is congruent with the idea that the brain is fundamentally affected in the illness, where widespread structural gray and white matter involvement, functionally abnormal cortical and subcortical information processing, and neurometabolic dysregulation are present in patients. Importantly, certain brain circuits appear preferentially affected and subtle abnormalities are already evident in first episode psychosis patients. We also demonstrated that brain circuitry alterations are clinically relevant by showing that these pathological signatures can be leveraged for predicting subsequent response to antipsychotic treatment. Interestingly, dopamine D2 receptor blockers alleviate neural abnormalities to some extent. Taken together, it is highly unlikely that the pathogenesis of schizophrenia is uniform, it is more plausible that there may be multiple different etiologies that converge to the behavioral phenotype of schizophrenia. Our data underscore that mechanistically oriented neuroimaging studies must take non-specific factors such as antipsychotic drug exposure or illness chronicity into consideration when interpreting disease signatures, as a clear characterization of primary pathophysiological processes is an imperative prerequisite for rational drug development and for alleviating disease burden in our patients.

## Introduction

Schizophrenia is a complex neuropsychiatric disorder with a diverse clinical phenotype that manifests in variable levels of positive and negative symptoms and cognitive impairment. Even though the course of the illness can be variable, for many patients the disease is chronic and debilitating, resulting in a substantial personal and public health burden (1). Available pharmacological interventions can alleviate positive symptoms, but effective treatments across symptom dimensions are lacking and a cure remains elusive. This can in part be attributed to the gaps in the knowledge of the underlying brain pathology. To close these gaps, tremendous efforts geared toward elucidating key pathophysiological brain signatures and at advancing our mechanistic understanding of the illness are undertaken with the ultimate goal to lower disease burden and improve long-term outcomes for patients.

**Figure 1:**
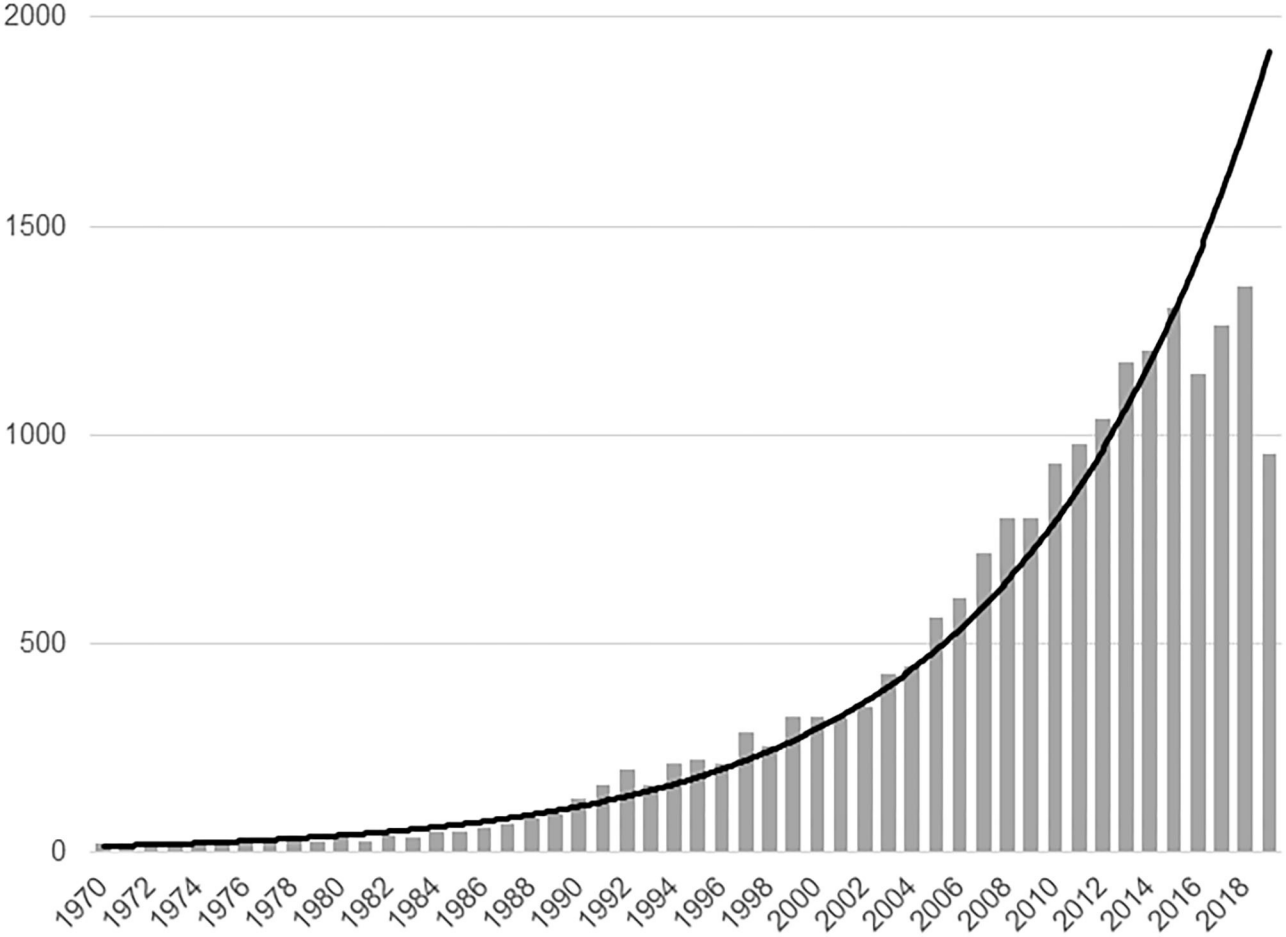
Neuroimaging studies in schizophrenia spectrum disorders. A PubMed search was performed on 7/29/20 with the following search term: (Schizophrenia OR Schizoaffective disorder OR Schizophreniform disorder OR Brief psychotic disorder OR Psychosis) AND (Neuroimaging OR PET OR SPECT OR MRI OR Diffusion OR Spectroscopy OR Connectivity). The search was restricted to studies published 1970 or later and to human subjects.

Neuroimaging offers versatility in terms of capturing different aspects of brain pathology *in vivo*, including brain structural integrity deficits, functional dysconnectivity, and altered neurotransmitter systems, positioning the field well to contribute to the discovery of clinically relevant biological processes in schizophrenia. The first mechanistic discovery studies utilizing neuroimaging date back to the 1970s and 80s, when ventricular enlargement was described as a potential diagnostic marker (2–4). Since these early studies, the field has grown exponentially; more than 16,000 neuroimaging manuscripts in schizophrenia spectrum disorder patients have been published (Figure 1). Even though major strides toward delineating relevant pathophysiological processes have been made, progress in the field has been impeded by a number of confounding factors that make interpretation of data difficult. This ultimately hampers the development of a comprehensive explanatory model capturing the complex underlying pathophysiology and integrating findings from diverse imaging modalities. Two principal confounding variables need to be considered when interpreting neuroimaging studies. These are antipsychotic medication exposure and illness chronicity, which impact virtually all imaging measures. This has resulted in a debate to what extent brain imaging alterations reported in schizophrenia studies reflect primary disease pathology (5). To make further progress, it is imperative to disentangle confounding effects from illness specific brain signatures. Investigating the impact of antipsychotic medications in longitudinal brain imaging studies and studying patients in the early illness stages who have had no prior antipsychotic medication exposure affords the opportunity to characterize antipsychotic drug action, mitigate confounds and gain a more in depth understanding of the nature of the illness.

Our group's work has been dedicated to contribute to the efforts in delineating the schizophrenia pathophysiology and in disentangling disease signatures from those of antipsychotic medications and disease chronicity. In the following paragraphs, we discuss a number of key scientific questions and take the opportunity to reflect on our progress toward advancing the mechanistic understanding of the illness.

## Key Scientific Questions in Neuroimaging Research in Schizophrenia

### What Can We Learn From Longitudinal Neuroimaging Studies That Investigate Antipsychotic Drug Effects?

Antipsychotic medications are the cornerstone of treating patients with schizophrenia spectrum disorders. They principally act on the dopamine D2 receptors (6), but also modulate a number of other neurotransmitter systems (7, 8), affect receptor expression profiles, have downstream effects on protein synthesis (9), and impact brain plasticity (9). Given the complexity of antipsychotic drug action, it is plausible that molecular imaging geared at investigating the dopamine system and other imaging markers are impacted by these medications. Longitudinal brain imaging studies that contrast neural signatures in the same patients before and after they are treated can shed light on modulatory effects of antipsychotic medications on brain structure, function and neurochemistry [for reviews of the larger field, see (10–15)]. We have conducted several studies utilizing different imaging techniques to broadly characterize antipsychotic drug effects on neural signatures (Table 1).

**Table 1:** Neuroimaging studies of antipsychotic drug effects from by the authors.

**Authors**	**Year**	**n (HC/SZ)**	**Treatment duration**	**Imaging marker**	**Region of interest**	**Baseline group differences**	**Effects of antipsychotic treatment on imaging marker**
**Structural MRI**							
Nelson et al. (16)	2018	23/37	6 weeks	Graph theory measures	Whole brain	HC< SZ in clustering, local efficiency and modularity	Baseline group differences in clustering, local efficiency, and modularity were no longer present after antipsychotic treatment
Nelson et al. (17)	2020	23/34	6 weeks	Cortical thickness, local gyrification index	Whole brain	HC> SZ in cortical thickness in the left brain hemisphere and global gyrification. HC> SZ in gyrification in the inferior frontal cortex, temporal cortex, insula, pre/postcentral gyri, temporoparietal junction, and supramarginal gyrus	Greater increase in cortical thickness after treatment was associated with a more favorable clinical response to treatment
**Diffusion imaging**							
Kraguljac et al. (18)	2019	42/42	6 weeks	Fractional anisotropy, mean diffusivity, radial diffusivity, radial fiber trophy	Whole brain	HC> SZ in fractional anisotropy in the medial temporal white matter, SZ> HC in mean diffusivity in the cingulum bundle	No change in microstructural diffusion indices or radial fiber trophy (surrogate marker of white matter volume)
Kraguljac et al. (19)	2019	42/42	6 weeks	Orientation dispersion index, extracellular free water	Whole brain	HC< SZ in orientation dispersion	No change in diffusion indices
**Task fMRI**							
Hutcheson et al. (20)	2015	20/21	1 week	BOLD response (episodic memory retrieval task), effective connectivity	11 regions of interest	HC< SZ in BOLD response in the middle cingulate gyrus during successful memory retrieval. HC> SZ in six effective connectivity paths, within frontal nodes, fronto-temporal projections and posterior to frontal projections. HC< SZ in four effective connectivity paths	Decrease in BOLD response in the middle frontal gyrus, postcentral gyrus, precuneus, superior parietal lobule, angular gyrus, and caudate. For effective connectivity, the number of edges increased after treatment. Five paths, within the frontal nodes, and frontal to posterior projections showed lesser connectivity after treatment, whereas 19 paths showed greater connectivity after treatment
Cadena et al. (21)	2018	20/22	6 weeks	BOLD response (Stroop task)	Whole brain	HC> SZ BOLD response in the ACC, caudate, putamen and midbrain	Increase in BOLD response in the ACC and caudate, decrease in BOLD response in the putamen, and midbrain
Cadena et al. (22)	2018	20/22	6 weeks	BOLD response (Stroop task)	Whole brain	HC> SZ BOLD response in the salience network and posterior default mode network	
Cadena et al. (23)	2019	20/22	6 weeks	Functional connectivity	Fronto-striatal network	HC< SZ in connectivity between the ACC, caudate and midbrain, HC> SZ between ACC and putamen	Decrease in connectivity between ACC, putamen, midbrain, and caudate
Gurler et al. (24)	2020	17/17	6 weeks	BOLD response (episodic memory encoding task)	Whole brain	HC> SZ in BOLD response in the right insula, hippocampus, inferior frontal and temporal cortex. SZ> HC in BOLD response in the PCC and precuneus	Increase in BOLD response in the hippocampus
**Resting state fMRI**							
Hadley et al. (25)	2014	21/21	1 week	Seed based functional connectivity, fALFF	Ventral tegmental area/midbrain (connectivity), whole brain (fALFF)	HC> SZ VTA connectivity to the ACC, superior, middle and inferior frontal gyri, insula, precuneus, inferior parietal cortex, thalamus, hippocampus, caudate, putamen and cerebellum. HC> SZ in fALFF in the ACC, MPFC, precuneus, PCC. HC< SZ in fALFF in the cerebellum	Ventral tegmental area/midbrain connectivity to the thalamus increased after treatment. fALFF increased in the dorsolateral prefrontal cortex and fronto-insular regions after treatment
Hadley et al. (26)	2016	32/32	6 weeks	Graph theory measures	Whole brain	HC< SZ in global clustering. HC> SZ in global efficiency	Global clustering decreased in good but not poor treatment responders. Global efficiency decreased in good but not poor responders
Kraguljac et al. (27)	2016	34/34	6 weeks	Seed based functional connectivity	Anterior and posterior hippocampus	HC< SZ in left anterior hippocampus connectivity with areas of the default mode network. HC> SZ in right anterior hippocampus connectivity to the PCC, precuneus, calcarine sulcus. HC> SZ in right posterior hippocampus connectivity to the ACC, supplemental motor cortex, inferior parietal cortex, PCC and precuneus	After treatment, connectivity between the right posterior hippocampus, ACC, and caudate increased, right anterior and posterior hippocampus connectivity to the lingual gyrus increased. Right anterior hippocampus connectivity to the auditory cortex and caudate decreased. Left anterior and posterior hippocampus connectivity to the auditory cortex decreased
Kraguljac et al. (28)	2016	34/34	6 weeks	Seed based functional connectivity	DMN, ECN, SN and DAN	HC< SZ in the ECN, SN, and DAN	DAN connectivity decreased after treatment
Lottman et al. (29)	2017	35/34	1 week, 6 weeks	ICA based static and dynamic functional connectivity	whole brain	HC< SZ within network connectivity in the cognitive control network and mixed increase and decrease in between network connectivity for static connectivity. SZ> HC in subcortical and somato-motor network connectivity in some instances in the sparsely connected state in dynamic connectivity	No change in static or dynamic connectivity after 1 or 6 weeks of treatment, respectively
**MRS**							
Cadena et al. (22)	2018	20/22	6 weeks	Glx	ACC	HC> SZ in Glx	No change in neurometabolites
Kraguljac et al. (30)	2019	31/61	6 weeks	Glx, NAA, Cho	ACC, hippocampus	HC< SZ in hippocampus Glx	No change in neurometabolites
Birur et al. (31)	2020	22/29	6 weeks, 16 weeks	Glx, NAA, Cho, Cr	MPFC	No group differences	No change in neurometabolites
**PET**							
Lahti et al. (32)	2003	0/6	12+/10 weeks (HAL); 23 ± 12 weeks (CLOZ)	rCBF	Whole brain	Not applicable	HAL and CLOZ increased rCBF in the ventral striatum and decreased rCBF in hippocampus and ventrolateral frontal cortex. The rCBF increase associated with HAL was greater than that with CLOZ in the dorsal and ventral striatum; the rCBF increase with CLOZ was greater than that with HAL in cortical regions, including the ACC and dorsolateral frontal cortex
Lahti et al. (33)	2004	12/6	12+/10 weeks (HAL); 23 ± 12 weeks (CLOZ)	rCBF	ACC	HC< SZ in rCBF during sensory motor control task HC> SZ in rCBF during decision task. No group differences at rest	CLOZ, but not HAL, reversed the abnormal ACC rCBF pattern in unmedicated patients
Lahti et al. (34)	2005	0/12	single dose	rCBF	Whole brain	Not applicable	rCBF increased in the striatum, thalamus, and ACC but decreased in frontal, temporal, and cerebellum regions after single dose HAL. rCBF increased in the ventral striatum, ACC, and temporal cortex but decreased in the thalamus and lingual cortex after single dose olanzapine. Both drugs activated the caudate nucleus
Lahti et al. (35)	2009	0/29	1 week, 6 weeks	rCBF	Whole brain	Not applicable	After 6 weeks of treatment both HAL and olanzapine increased rCBF in the pre- and post-central cortex, ventral striatum, and caudate, and decreased rCBF in the anterior cingulate and medial frontal cortex
Bolding et al. (36)	2012	0/29	1 week, 6 weeks	rCBF, functional connectivity	Whole brain, ROI analysis	Not applicable	rCBF increased in the striatum and decreased in the MPFC after 1 week of treatment. rCBF increased in the thalamus and decreased in the putamen, hippocampus, and middle frontal cortex between week one and six of treatment. Functional connectivity between the middle frontal cortex and nucleus accumbens increased after 1 week of treatment, connectivity between the middle frontal cortex and hippocampus decreased after 6 weeks of treatment
**Multimodal imaging**							
Cadena et al. (22)	2018	20/22	6 weeks	ACC Glx, BOLD response (Stroop task)	Whole brain	Glx and BOLD response in the salience network and posterior default network correlated in HC but not SZ at baseline	After treatment, the relationship between Glx and BOLD response changed direction in both groups
Gurler et al. (24)	2020	17/17	6 weeks	hippocampus Glx, BOLD response (memory encoding task)	Whole brain	In SZ but not in HC, hippocampal Glx and BOLD response the posterior DMN correlated. The relationship between BOLD signal in the DMN and hippocampal Glx was significantly different between groups	After treatment, the correlation between Glx and BOLD response was not significant and was not significantly different from HC

*ACC, anterior cingulate cortex; BOLD, Blood oxygen dependent level: Cho, choline; CLOZ, clozapine; Cr, Creatine; DAN, dorsal attention network; DMN, default mode network; ECN, executive control network; fALFF, fraction of the amplitude of low frequency fluctuations; Glx, glutamate+glutamine; HAL, haloperidone; HC, Healthy control; ICA, independent component analysis; MPFC, medial prefrontal cortex; NAA, N-acetyl-aspartate; PCC, posterior cingulate cortex; rCBF, regional cerebral blood flow; ROI, region of interest; SN, salience network; SZ, patients with schizophrenia*.

Our structural imaging studies show mixed results in terms of antipsychotic medication effects. We observed antipsychotic medication related changes in cortical thickness (17), but not in white matter microstructural integrity or radial fiber trophy (a surrogate marker for white matter volume) (18, 19). The disparity in effects across tissue classes is perhaps not surprising, given the different anatomical, functional and neurochemical makeup of these tissues. For example, brain derived neurotrophic factor (BDNF) is a neurotrophin that regulates synaptic plasticity (37, 38) and spine density (39). The 66met allele has been associated with diminished activity-dependent BDNF secretion (40) and decreased cortical volumes (41) but not with white matter integrity in healthy subjects (42). BDNF also is found to be abnormally low in schizophrenia (43) and can be increased by second generation antipsychotics (44). It is therefore possible that antipsychotic medication related modulation of BDNF results in alleviation of dendritic deficits in the outer layers of the cortex (45), which would be reflected in an increase in cortical thickness, but not white matter integrity. Alternatively, it is possible that the acute gray matter tissue response can be captured at the time scale at which we investigated structural changes, but the duration is not sufficient to demonstrate subtle changes in white matter architecture and microstructure that may become more evident with longer duration of antipsychotic treatment.

Our longitudinal task based and resting state functional neuroimaging studies consistently show changes associated with antipsychotic drugs at different time scales, ranging from a single dose drug administration to several weeks of treatment. Our findings are in agreement with drug challenge studies in healthy subjects demonstrating that dopamine antagonism affects brain function (46) and animal studies showing that antipsychotics can be used to restore cortical synchronization and functional connectivity after disruption with hallucinogenic agents (47, 48). When interpreting our functional data in context of differences between patients and healthy controls, we show that antipsychotic treatment appears to, at least partially, restore abnormal brain function to levels that are more similar to those of healthy controls. We also observed a recurring theme of brain regions that are functionally modulated by antipsychotic treatment. Relevant areas include the anterior cingulate cortex, medial prefrontal cortex, hippocampus, caudate and putamen. This suggests that cortico-striatal circuitry may be functionally remodeled by antipsychotic medications. Interestingly, anterior cingulate and medial frontal cortex function (35, 49–52) and cortico-striatal connectivity have also been identified as critical for antipsychotic drug action by other groups (53, 54). When examining brain function on a more global level, we found that antipsychotic medications affect brain network topology, i.e., global efficiency and clustering, but not dynamic connectivity states (26, 29), which is consistent with the idea that antipsychotic medications partially but not fully restore abnormal brain function.

In our magnetic resonance spectroscopy studies, we unexpectedly did not find changes with antipsychotic medications on glutamate levels. Several lines of evidence describe complex interactions between the dopamine and glutamate systems and identify glutamate receptor complexes as potentially important indirect targets for dopamine D2 receptor blockers (55–57), suggesting antipsychotic medications may affect glutamate levels. A number of prior studies report a decrease of glutamate in the temporal lobe (58), striatum, and the anterior cingulate cortex (59), though the group failed to replicate the latter in a subsequent report (60). It is possible that this discrepancy is due to differential effects of various antipsychotic medications on the glutamate system. Alternatively, risperidone may affect glutamate levels only in a subset of patients, or the shifts in glutamatergic neurotransmission may be too subtle to be captured with magnetic resonance spectroscopy, which measures tissue metabolites rather than compartment specific levels.

Our data also suggest that heterogeneity in the patient population may need to be taken into account when examining antipsychotic medication related changes in neural signatures. We demonstrated in several studies that changes in brain structure and function following antipsychotic treatment may be more prominent in patients with a favorable clinical response, compared to those who show little improvement with medications (17, 26, 27). It is tempting to speculate that the variability in modulatory effects of antipsychotics is reflective of differences in brain pathology. It is possible that patients who respond favorably to medications do so because they still have actively destructive, yet reversible, neurobiological changes that can be ameliorated with antipsychotic treatment. In contrast, poor responders may be beyond the period of active deficit process formation in their brain and the pathological state becomes more permanent, rendering antipsychotic medications ineffective both on a clinical level and in reversing structural and functional brain injury. If that is the case, it should be possible to leverage neuroimaging data to aid in the prediction of response to antipsychotic treatment. Much of the work described here has been performed in patients who had prior antipsychotic medication exposure, and in mixed groups of antipsychotic medication-naïve patients previously medicated patients. Adequately powered longitudinal studies conducted in exclusively medication-naïve patients are direly needed to reduce the possible effects of illness chronicity and prior antipsychotic medication exposure on changes related to antipsychotic medication treatment.

### Can Neuroimaging Data Aid in Predicting Subsequent Clinical Response to Antipsychotic Treatment?

The current strategy for management of psychosis spectrum disorders consists of sequential treatment with different antipsychotic medications based on trial and error. Unfortunately, it is not possible to predict how a patient will respond to treatment based on clinical assessments alone. To make progress in this context, we have conducted a number of studies geared toward characterizing neural signatures in unmedicated and antipsychotic medication-naïve patients that predict a subsequent favorable response to antipsychotic treatment (Table 2). Here, the ultimate goal is to realize the potential of neuroimaging in guiding treatment decisions based on the underlying brain pathology [for further review of the topic, see (63, 64)].

**Table 2:** Selected baseline neuroimaging markers that are relevant to the prediction of subsequent antipsychotic drug response.

**Authors**	**Year**	***n***	**Illness stage**	**Duration of treatment**	**Imaging marker**	**Association between baseline imaging findings and subsequent antipsychotic treatment response**
**Structural MRI**						
Hutcheson et al. (61)	2014	23	mixed	6 weeks	Regional subcortical brain volume	Higher volumes of the caudate, putamen, and pallidum predicts better antipsychotic treatment response.
Nelson et al. (17)	2020	34	Mixed	6 weeks	Cortical thickness, local gyrification index	Lower cortical thickness in the prefrontal cortex, pre- and post-central gyri, cingulate, and insula is associated with favorable subsequent response to treatment. Gyrification index is not predictive of treatment response.
**Diffusion imaging**						
Kraguljac et al. (62)	2020	66	First episode	16 weeks	Fractional anisotropy, mean diffusivity, radial diffusivity, axial diffusivity	Lower whole brain baseline fractional anisotropy predicts poorer antipsychotic treatment response.
Kraguljac et al. (19)	2019	42	Mixed	6 weeks	Orientation dispersion index, extracellular free water	Higher baseline whole brain orientation dispersion index predicts poorer antipsychotic treatment response.
**Task fMRI**						
Cadena et al. (22)	2018	22	Chronic	6 weeks	BOLD response (Stroop task)	Greater BOLD response in the striatum and midbrain predicts better antipsychotic treatment response.
Cadena et al. (23)	2019	22	Chronic	6 weeks	Functional connectivity	Greater connectivity between the ACC and putamen predicts better antipsychotic treatment response.
**Resting state fMRI**						
Hadley et al. (25)	2014	21	Mixed	1 week, 6 weeks	Seed based functional connectivity of the ventral tegmental area, whole brain fALFF	Baseline ventral tegmental area connectivity to the dorsal ACC and supplemental motor cortex was positively correlated with treatment response. Baseline connectivity between the ventral tegmental area and the DMN was negatively correlated with treatment response.
Kraguljac et al. (27)	2016	34	Mixed	6 weeks	Seed based functional connectivity of the hippocampus	Baseline connectivity between the hippocampus, ACC, caudate nucleus, auditory cortex, and calcarine sulcus predicts subsequent antipsychotic treatment response.
Kraguljac et al. (28)	2016	34	Mixed	6 weeks	Seed based functional connectivity of the DMN, ECN, SN, and DAN	Baseline connectivity of the DAN to the cuneus, precuneus, superior and inferior parietal lobes, lingual gyrus, middle occipital lobe, and calcarine sulcus is positively correlated with antipsychotic treatment response.
**MRS**						
Kraguljac et al. (30)	2019	61	Mixed	6 weeks	Glx, NAA, Cho	Lower NAA in the ACC and higher Glx in the hippocampus at baseline were associated with better response to treatment at trend level.
**PET**						
Lahti et al. (35)	2009	29	Chronic	1 week, 6 weeks	rCBF	Greater increase in rCBF in the ventral striatum and greater decrease in rCBF in the hippocampus after 1 week of treatment was associated with a good response to antipsychotic medication after 6 weeks of treatment.
Bolding et al. (36)	2012	29	Chronic	1 week, 6 weeks	Functional connectivity	Functional connectivity between the middle frontal cortex and left hippocampus after 1 week of treatment predicted response to antipsychotic treatment after 6 weeks.

*ACC, anterior cingulate cortex; BOLD, blood oxygen dependent level; CEN, central executive network; Cho, Choline; DAN, dorsal attention network; DMN, default mode network; fALFF, fractional amplitude of low frequency fluctuations; Glx, glutamate+glutamine; NAA, N-acetyl-aspartate; Rcbf, regional cerebral blood flow; SN, salience network*.

In structural imaging studies, we found that both gray and white matter integrity at baseline are predictive of subsequent response to antipsychotic treatment. Lower cortical thickness and greater basal ganglia volumes [basal ganglia volumes are increased in patients, possibly as a result of antipsychotic drug exposure (65)] are associated with better treatment response (17, 61). This suggests that greater baseline alterations in gray matter are indicative of a favorable clinical outcome. In contrast, greater alterations in whole brain fractional anisotropy, a non-specific marker of white matter integrity, and in whole brain orientation dispersion, a measure of fiber complexity, were associated with subsequent poor treatment response, suggesting that greater alterations in white matter are indicative of poor clinical outcomes (18, 19, 62).

Our functional neuroimaging studies, both positron emission tomography (PET) and functional MRI, found consistent associations between more intact brain function at baseline and a favorable subsequent response to antipsychotic medications. Here again we found a recurring theme of brain regions and networks that were relevant for clinical outcomes, specifically anterior cingulate cortex, hippocampus, thalamus, striatum and caudate, as well as the default mode network and dorsal attention network. Interestingly, we also found that the degree of normalization in brain function after 1 week of antipsychotic medication was predictive of clinical outcomes after 6 weeks of treatment (35, 36). Our data suggest that both baseline function and early functional changes may be important predictors of treatment response.

Our magnetic resonance spectroscopy studies investigated neurometabolite levels in the anterior cingulate cortex and the hippocampus (30). We found that N-acetyl-aspartate (NAA), a marker of neuronal health, in the anterior cingulate cortex and glutamate in the hippocampus at baseline were associated with response to risperidone after 6 weeks of treatment. At first glance, the association between low cortical NAA and good response to treatment appears counter-intuitive. It is striking, however, that greater alterations in both NAA and glutamate are linked to better treatment response. One could speculate that these features point toward an excitation/inhibition imbalance and a deprived neuronal state that is reversible by successful antipsychotic treatment.

Taken together, our studies suggest two groups of neuroimaging markers that are relevant for subsequent antipsychotic treatment response. The first is a set of markers for which greater abnormalities predict subsequent poor response to antipsychotic treatment. These markers are largely centered around the theme of brain connectivity, where greater abnormal functional connectivity and white matter integrity deficits are indicative of worse response to antipsychotic treatment. The second is a set of markers where greater abnormalities predict a favorable response to antipsychotic treatment. These markers include cortical thickness, subcortical brain volumes and neurometabolites levels. Abnormalities in these imaging features may signify a deprived neuronal state or a disequilibrium in the excitation/inhibition balance that may be reversible with successful antipsychotic treatment. Overall, our findings suggest that the brain is structurally and functionally “wired” in a way that does or does not favor response to antipsychotic medication treatment. However, based on these studies, it remains unclear if the neural signatures relevant for antipsychotic treatment response are also core features of the schizophrenia pathophysiology or not. To disentangle core illness features from confounds of antipsychotic medication exposure as well as illness chronicity, it is imperative to characterize neuroimaging signatures in antipsychotic medication-naïve first-episode psychosis patients.

### What Are the Neuroimaging Signatures That Reflect the Core Pathology in Schizophrenia?

There is a growing sense that progressive brain changes occur beyond the first psychotic episode and that illness chronicity may fundamentally affect the brain (66). It is difficult to pinpoint the extent to which abnormalities can be attributed to antipsychotic medication effects or to ongoing disease progression. Studying patients at later illness stages makes it problematic to discern downstream effects of pathology and prior treatment attempts from primary pathology. Even though this patient population is difficult to recruit (67), studying antipsychotic medication-naïve first episode psychosis patients can mitigate these issues. Our group has conducted studies in this population using different imaging modalities in an effort to characterize relevant neural signatures (Table 3).

**Table 3:** Neuroimaging studies of antipsychotic medication-naïve first episode psychosis patients from our group.

**Authors**	**Year**	***n* (HC/FEP)**	**Imaging marker**	**Region of Interest**	**Group differences**
**Structural MRI**					
Maximo et al. (68)	2019	0/55	Gray matter volume, cortical thickness, cortical surface area	DMN, CEN, SN	Greater duration of untreated psychosis was associated with reduced surface area in the salience network and central executive network. Cortical thickness and duration of untreated psychosis were correlated in the SN and DMN.
Briend et al. (69)	2020	40/40	Gray matter volume	Hippocampus subfields	HC> FEP in whole hippocampus volume, as well as volume of the CA1, molecular layer, subiculum, presubiculum, and hippocampal tail.
Nelson et al. (17)	2020	23/22	Cortical thickness, gyrification index	Whole brain	HC> FEP in gyrification in the precentral and postcentral gyri, and at trend level in cortical thickness.
**Diffusion imaging**					
Kraguljac et al. (18)	2019	42/30	Fractional anisotropy, mean diffusivity, radial diffusivity	Whole brain	HC> FEP in fractional anisotropy in the medial temporal lobe. HC< FEP in mean diffusivity in the hippocampal part of the cingulum bundle.
Kraguljac et al. (19)	2019	42/30	Orientation dispersion index, extracellular free water	Whole brain	HC< FEP in orientation dispersion in the posterior limb of the internal capsule. No group differences in extracellular free water.
Kraguljac et al. (62)	2020	45/66	Fractional anisotropy, mean diffusivity, radial diffusivity, axial diffusivity	Whole brain	HC> FEP in whole brain fractional anisotropy and axial diffusivity.
**Resting state fMRI**					
Maximo et al. (68)	2019	0/55	Seed based functional connectivity	DMN CEN, SN	Longer duration of untreated psychosis was associated with reduced connectivity in all three networks and with a single cluster of increased connectivity in the CEN.
Briend et al. (70)	2020	40/40	Static and dynamic connectivity (quasi-periodic patterns)	Fronto-parietal network	HC> FEP in static connectivity. The strength of the correlation of quasi-periodic patterns in the quasi-periodic pattern sliding vector was stronger in FEP than in HC, suggesting a greater impact on intrinsic brain activity in FEP. Regressing quasi-periodic patterns from functional scans erased connectivity group differences, suggesting that abnormal connectivity in FEP could result from altered quasi-periodic patterns.
Nelson et al. (71)	2020	41/55	Seed based functional connectivity	Hippocampus	HC< FEP in hippocampus connectivity to the cuneus, precuneus, and lingual gyrus. HC> FEP in hippocampus connectivity to the PCC, parahippocampus, and rostral orbitofrontal cortex.
**MRS**					
Sivaraman et al. (72)	2018	18/14	Glx, NAA, Cho	Associative striatum	HC< FEP in choline levels, relationship between NAA and Glx is disrupted in patients.
Birur et al. (31)	2020	22/29	Glx, NAA, Cho, Cr	MPFC	No group differences
Nelson et al. (71)	2020	41/55	Glx, NAA	Hippocampus	No group differences
Briend et al. (69)	2020	41/54	Glx	Hippocampus	HC< FEP in Glx in patients with long but not short duration of untreated psychosis.
**Multimodal imaging**					
Nelson et al. (71)	2020	41/55	Glx, seed based functional connectivity	Hippocampus	Greater hippocampal connectivity was correlated with higher Glx levels in the anterior and posterior DMN in both groups. In the entorhinal and orbitofrontal cortices, higher Glx levels predicted greater connectivity in HC, and the opposite was true in FEP.
Briend et al. (69)	2020	41/54	Glx, subfield volumes	Hippocampus	No associations between Glx and hippocampus subfield volumes in both groups.

*CEN, central executive network; Cho, Choline; Cr, Creatine; DMN, default mode network; FEP, first episode psychosis patients; Glx, glutamate+glutamine; HC, healthy control; NAA, N-acetyl-aspartate; SN, salience network*.

Using structural neuroimaging techniques, we found that a number of alterations are already present in medication-naive patients. Alterations include decreased gyrification in the pre- and post-central gyri, decreased total hippocampus and hippocampus subfield volumes, a trend level decrease in cortical thickness, as well as a decrease in whole brain fractional anisotropy and axial diffusivity (17, 18, 62, 69). Additionally, an increase in mean diffusivity in the hippocampal part of the cingulum, which is often interpreted as evidence of neuroinflammation, is already evident in first episode patients (18). Importantly, the duration of untreated psychosis, which is the time between first onset of psychotic symptoms and the first antipsychotic treatment, seems to be an important modulatory factor for structural integrity, as a longer duration of untreated psychosis is associated with greater gray and white matter alterations (62, 68, 69).

We saw similar results in our functional imaging studies where greater duration of untreated psychosis was associated with greater disruptions in functional connectivity in large-scale brain networks supporting higher order cognition (68). In the overall group of antipsychotic medication-naïve patients, we note that hippocampus connectivity is disrupted. Spatial patterns of dysconnectivity resemble those in unmedicated, chronic schizophrenia patients (71). When examining dynamic connectivity, we found that quasi-periodic patterns had a greater impact of fronto-parietal control network connectivity in first episode patients compared to controls, suggesting that brain network dynamics are already altered in this patient group (70). Taken together, connectivity alterations are clearly already present in medication-naïve patients, which is consistent with the hypothesis that brain network dysconnectivity is a core feature of the illness (73–75).

In magnetic resonance spectroscopy studies, we found elevated choline and a disruption of the relationship between NAA and glutamate in the left striatum, indicating possible mitochondrial, membrane, and glial dysfunction (72). However, we reported no neurometabolite abnormalities in the medial prefrontal cortex or the hippocampus when contrasting antipsychotic medication-naïve first episode patients and controls in the overall group (31, 69). It is important to note that glutamate levels in both areas showed a greater variance in patients compared to controls, suggesting heterogeneity in glutamatergic metabolism in first episode psychosis. When the duration of untreated psychosis is taken into consideration, those with a shorter but not those with a longer duration of untreated psychosis did have an elevation in hippocampal glutamate (69).

Collectively, it is evident that functional and structural abnormalities are already present in patients who are in their early illness stages who have no prior exposure to antipsychotic medications. However, wee did not observe group level alterations in NAA or glutamate which was somewhat unexpected. Interestingly, the variance in measurements was greater in patients compared to controls, suggesting that neurometabolite alterations may only be present in a subset of patients, or become more pronounced as the illness progresses. Importantly, we found the duration of untreated psychosis to be an important mediator, such that a longer interval adversely impacts brain structure and function, underscoring the importance of early intervention efforts in this syndrome.

### Can Multimodal Brain Imaging Help Us Better Characterize the Complex Pathophysiology in Schizophrenia?

The field is moving from traditional models of schizophrenia focused on the disruption of a single molecule such as dopamine or glutamate, to models of pathway dysregulation as a means of integrating findings from diverse imaging modalities. An individual component of a pathway in this context can be best understood as part of a highly interactive network that may influence other parts of the network and in turn be regulated by a number of other factors (76). An abnormality may represent a primary etiological factor, but could also be present because of a disruption in modulatory inputs and thus reflect a secondary consequence of a pathophysiological process. It is critical to develop disease models with a degree of complexity that accurately reflect these highly interactive patterns. Multimodal imaging allows investigation of brain dysfunction using a range of techniques within the same individual and to test hypotheses about relationships between biological mechanisms. This approach may reveal details about the pathophysiology that would not be detectable when using one modality alone.

For example, glutamatergic hyperactivity is hypothesized to be a key pathological feature in schizophrenia (77, 78). Glutamate neurotransmitter flux, neuronal firing rate, and the blood oxygen level dependent (BOLD) response are tightly coupled (79, 80), and glutamate plays a role in long-range functional connections (81). In preclinical studies, excess glutamate has been shown to be associated with disorganized neuronal activity (82) and may result in increased synapse turnover as well as axonal or glial injury (83–85). If this premise holds true, multimodal neuroimaging studies should detect relationships between glutamate levels and brain function in healthy subjects that are disrupted in patients with schizophrenia. They also should reveal an association between glutamate excess and structural integrity deficits in patients. Our group has conducted a number of such studies to empirically test the relationship between glutamate and brain function the impact of glutamate excess on brain structure.

We used several task paradigms and resting state functional MRI to characterize the relationship between glutamate levels and brain function in healthy subjects and schizophrenia spectrum patients. We reported a positive correlation between hippocampal glutamate and inferior frontal activation during a memory retrieval task in controls that was absent in medicated chronic schizophrenia patients (86). The relationship between hippocampal glutamate and the BOLD response in default mode network regions during memory encoding also differed between unmedicated patients and controls (24). Using a Stroop task, we observed a negative correlation between anterior cingulate cortex glutamate and the BOLD response in the posterior cingulate cortex, precuneus, occipital cortex and cerebellum in controls; this correlation was inverted in medicated first-episode psychosis patients (87). Similarly, we noted a correlation between anterior cingulate cortex glutamate and the BOLD response in the salience network and posterior default network during a Stroop task in healthy controls but not unmedicated patients with schizophrenia (22). Using a reward task, we observed an association between the prediction error related BOLD response in the midbrain and substantia nigra glutamate in healthy controls but not medicated patients with chronic schizophrenia. Interestingly, glutamate levels were elevated in patients, suggesting that glutamatergic dysfunction might contribute to abnormal neural prediction error coding (88). At rest, we initially did not detect a relationship between hippocampal glutamate and aberrant hippocampal connectivity to the precuneus in a small group of unmedicated schizophrenia patients (89). However, after expanding the sample size, we did observe that higher glutamate levels were correlated with higher hippocampus resting state connectivity to the anterior default mode network in healthy controls, but the relationship between measures was inverse in first episode psychosis patients (71). Taken together, our studies consistently demonstrated a link between glutamate levels and brain function, regardless of task condition and even in absence of a task in healthy subjects. Importantly, this relationship was consistently found to be altered or absent in patients across paradigms tapping into various aspects of brain function, which implicates that the disruption in this coupling may be a fundamental feature of the illness. Interestingly, a detectable alteration in glutamate levels does not seem to be a necessary element to reach the threshold of a disruption in brain function, suggesting the presence of subtle abnormalities in glutamatergic neurotransmission that may be below the level of detection with magnetic resonance spectroscopy. Our longitudinal studies further demonstrate that the relationship between glutamate and brain function in patients is modulated by antipsychotic drug treatment, suggesting a potential mechanism of antipsychotic drug action (22, 24).

We also conducted studies to examine the impact of glutamate (excess) on brain structures. In unmedicated chronic schizophrenia patients, we found that higher hippocampal glutamate was associated with lower hippocampal volumes, suggesting that glutamate related excitotoxicity (neurotransmitter excess related increased synapse turnover) might affect brain structure (90). Our finding is in agreement with a later study that reported a negative relationship between glutamate excess and brain volumes in the caudate nucleus in first episode psychosis patients (91). In contrast, we did not observe a linear relationship between hippocampus subfield volumes and glutamate levels in antipsychotic medication-naïve first episode psychosis patients (69). Interestingly though, those with a longer duration of untreated psychosis showed lower hippocampus subfield volumes. While this discrepancy between our studies at first glance does not support the hypothesis that glutamate impacts brain structures as a fundamental mechanism of the illness, it is possible that the amount of time the brain is exposed to altered glutamate is the key factor in adversely affecting brain structures rather than the amount of glutamate present in the brain at any given time.

As demonstrated with the above examples, multimodal neuroimaging clearly can give important insights into the pathophysiology of the illness, and allows testing of hypotheses about the relationships between biological mechanisms which is not possible with a single imaging modality. However, because different aspects of the pathophysiology are typically assessed at the same time in multimodal imaging, interpretations are limited to discovery of associations but not causality. In other words, it is possible to detect a relationship between two different imaging markers, but it is not feasible to discern if one abnormality is caused by the other or vice versa.

### Can Pharmacological Challenge Studies Provide a Framework for the Interpretation of Neuroimaging Findings in Schizophrenia?

A complementary line of research, pharmacological challenge studies, which are designed to model aspects of the pathophysiology, can inform inferences drawn about causality for neuroimaging alterations seen in schizophrenia spectrum patients. Our group has done several pharmacological challenge studies using subanesthetic ketamine, a non-competitive N-methyl-d-aspartate (NMDA) receptor blocker that transiently induces a behavioral phenotype similar to that seen in the illness (92–96), to test the hypothesis that experimentally induced NMDA receptor hypofunction causes changes in brain function and neurochemistry that are comparable to neural signatures observed in schizophrenia.

Early imaging studies testing the effects of a pharmacological challenge found that sub-anesthetic ketamine affects regional cerebral blood flow (rCBF). In response to the drug, an increase in psychosis symptom severity was paralleled by an increase in rCBF in the anterior cingulate cortex, both in healthy controls and medicated patients with schizophrenia (97). In addition to a ketamine related increase in rCBF in the anterior cingulate cortex, a reduction in rCBF was found in the hippocampus, lingual gyrus and fusiform gyrus in a different study of healthy controls (98). Interestingly, kinetic analyses suggest that the ketamine induced rCBF response differs across brain areas (99). Using a MRI based rather than PET based technique to quantify blood flow, our group later reported increased rCBF in the prefrontal cortex, cingulate cortex caudate, putamen, thalamus and hippocampus, as well as interregional connectivity alterations in areas of the salience network (100). This again suggests systems level, but not uniform, effects of experimentally induced NMDA receptor hypofunction.

In a combined magnetic resonance spectroscopy and resting state functional connectivity study of the hippocampus (101), we reported that experimentally induced NMDA receptor hypofunction resulted in an increase in glutamate that was similar in magnitude to that we saw in unmedicated schizophrenia patients (90). In parallel, we also found a reduction in hippocampal resting state connectivity to the anterior cingulate cortex, medial prefrontal cortex, insula, hippocampus, precuneus, posterior cingulate cortex and lingual gyrus. Spatial patterns of abnormalities here closely resemble those we observed in both unmedicated schizophrenia patients (27, 71) and antipsychotic medication-naïve first episode psychosis patients (71).

Taken together, our findings from ketamine challenge studies lend empirical support to the putative link between NMDA receptor hypofunction, disruption in brain function and glutamate excess and provide a theoretical framework for the interpretation of abnormal brain signatures in schizophrenia in the context of NMDA receptor hypofunction. It is important to note that this type of framework can also be leveraged to test target engagement for putative novel pharmacological agents as recently demonstrated a multi-center proof of mechanism study of pomaglumetad (102), which will ideally translate into accelerated development of novel drugs.

### How Can Postmortem Work Provide Context for the Neuroimaging Findings in Schizophrenia?

Postmortem studies reporting abnormalities in γ-Aminobutyric-acid (GABA)ergic interneurons (103, 104) and glutamatergic signaling (105) provided the impetus for the measurement of glutamate and GABA using MR spectroscopy, as well as the measurement of brain oscillations using EEG/MEG. The fast-spiking GABA-interneurons play a fundamental role in controlling of the synchrony of cortical pyramidal neurons by producing rhythmic inhibitory postsynaptic potentials. Consequently, these interneurons appear to be key to the generation of gamma oscillations (106). In a group of first episode psychosis patients scanned using an ultra-high field magnet [7 Tesla (T)] we reported a decrease in glutamate, but not GABA levels, in the anterior cingulate cortex (107). Interestingly, in a larger group of first episode psychosis patients also scanned at 7T, Wang reported both a decrease in anterior cingulate glutamate and GABA levels (108). In the same group of first episode psychosis patients in which we found a glutamate decrease (107), we did not find abnormalities in gamma range oscillations using MEG (109), although others, also in first episode patients, have reported such alterations (110–112). All three of these studies used EEG, and it has been shown that there can be differences in the cortical auditory evoked response between MEG and EEG (113). Inspired by imaging studies reporting decrease in N-acetyl-aspartate, a marker of neuronal integrity, in the anterior cingulate cortex in schizophrenia (114), Roberts (115) used electron microscopy in anterior cingulate cortex postmortem brain samples and identified a decrease in the number of excitatory synaptic connections, as well as a decrease in the number of mitochondria per neuronal somata, suggesting a decrease in cortical efficiency in schizophrenia.

### How Does Neuroimaging Inform Our Mechanistic Understanding of the Illness?

Our work has contributed to the extensive efforts in studying the structural and functional neuroanatomy of schizophrenia by mapping relevant neural signatures in psychosis spectrum patients. Our data is congruent with the idea that the brain is fundamentally affected in the illness, where widespread structural gray and white matter involvement, functionally abnormal cortical and subcortical information processing, and neurometabolic dysregulation are present in patients. Importantly, data indicate that pathology is not merely diffusely distributed across the entire brain, rather it appears that certain brain circuits are preferentially affected. The evidence is compelling that many of the subtle abnormalities described in chronic schizophrenia are already evident in first episode psychosis patients, highlighting that these brain signatures are likely to be relevant to the core pathology and not a just be a consequence of other non-specific factors associated with the illness (116). We also demonstrated that these brain circuitry alterations are clinically relevant by showing that these pathological signatures can be leveraged for predicting subsequent response to antipsychotic treatment, further underscoring that they are key features of the illness.

Despite these successes in delineating disease signatures, it remains challenging to identify casual factors leading to these alterations through cross-sectional unimodal mapping alone (117). To gain further insights, our group has leveraged different approaches. First, we used various pharmacological challenges to characterize the effects of active modulation of major neurotransmitter systems on neural signatures. We clearly demonstrated that dopamine D2 receptor blockers alleviate neural abnormalities to some extent, and that experimentally induced NMDA receptor blockage results in alterations that resemble those seen schizophrenia, underscoring the pathophysiological relevance of these neurotransmitter systems. Second, we combined findings from different imaging modalities to gain additional insights into the role of glutamatergic neurotransmission for the modulation of functional brain networks. Our studies consistently demonstrated a disruption in the link between glutamate levels and brain function in patients across paradigms tapping into various aspects of brain function, which implicates that the disruption in this coupling may be a fundamental feature of the illness. Third, postmortem studies can also inform mechanistic understanding. For example, decrease in mitochondrial function observed in schizophrenia postmortem samples (115) suggest abnormalities in brain bioenergetics.

Taken together, it is highly unlikely that the pathogenesis of schizophrenia is uniform (118), it is more plausible that there may be multiple different etiologies that converge to the behavioral phenotype of schizophrenia (119). Our data underscore that mechanistically oriented neuroimaging studies must take non-specific factors such as antipsychotic drug exposure or illness chronicity into consideration when interpreting disease signatures, as a clear characterization of primary pathophysiological processes is an imperative prerequisite for rational drug development and for alleviating disease burden in our patients.

## Author Contributions

AL and NK wrote the manuscript. Both authors contributed to and approved the final version of this manuscript.

## Conflict of Interest

NK serves as consultant for Neurocrine Biosciences, Inc. The remaining author declares that the research was conducted in the absence of any commercial or financial relationships that could be construed as a potential conflict of interest.
